# Raffinose, a plant galactoside, inhibits *Pseudomonas aeruginosa* biofilm formation via binding to LecA and decreasing cellular cyclic diguanylate levels

**DOI:** 10.1038/srep25318

**Published:** 2016-05-04

**Authors:** Han-Shin Kim, Eunji Cha, YunHye Kim, Young Ho Jeon, Betty H. Olson, Youngjoo Byun, Hee-Deung Park

**Affiliations:** 1 School of Civil, Environmental and Architectural Engineering, Korea University, Anam-Dong, Seongbuk-Gu, Seoul 136-713, South Korea; 2 College of Pharmacy, Korea University, Sejong-ro 2511, Jochiwon-eup, Sejong, 339-700, South Korea; 3 Department of Civil and Environmental Engineering, University of California, Irvine, CA 92697, USA

## Abstract

Biofilm formation on biotic or abiotic surfaces has unwanted consequences in medical, clinical, and industrial settings. Treatments with antibiotics or biocides are often ineffective in eradicating biofilms. Promising alternatives to conventional agents are biofilm-inhibiting compounds regulating biofilm development without toxicity to growth. Here, we screened a biofilm inhibitor, raffinose, derived from ginger. Raffinose, a galactotrisaccharide, showed efficient biofilm inhibition of *Pseudomonas aeruginosa* without impairing its growth. Raffinose also affected various phenotypes such as colony morphology, matrix formation, and swarming motility. Binding of raffinose to a carbohydrate-binding protein called LecA was the cause of biofilm inhibition and altered phenotypes. Furthermore, raffinose reduced the concentration of the second messenger, cyclic diguanylate (c-di-GMP), by increased activity of a c-di-GMP specific phosphodiesterase. The ability of raffinose to inhibit *P. aeruginosa* biofilm formation and its molecular mechanism opens new possibilities for pharmacological and industrial applications.

Most bacteria are able to live in two different states: planktonic and sessile. A biofilm is an assemblage of a single or multiple species that are encapsulated in self-produced extracellular polymeric substances (EPS). Notably, bacterial infections are frequently associated with biofilms^1^. For example, cystic fibrosis patients infected with *Pseudomonas aeruginosa* show a progressive loss of pulmonary function due to its biofilm formation in the lung^2^. Moreover, biofilms tend to foul medical devices, contact lens, and artificial implants, which eventually lead to critical medicinal problems^3^,^4^. Beyond these, biofilms in the industrial settings (e.g. ship hulls, water pipes, and membrane filters) cause loss of performance and increased cost for maintenance and quality control^5^. Nevertheless, biofilms formed on biotic and abiotic surfaces are notoriously difficult to eradicate, mainly because biofilm cells are insensitive to antimicrobial agents or biocides^6^. Biofilm cells are known to be 10–1,000 fold more resistant to antimicrobial agents than planktonic cells^3^.

In contrast to traditional bactericidal or bacteriostatic approaches to inhibit biofilms, a recently developed control method is targeted at interference with biofilm development without affecting bacterial growth^7^. For example, bacterial cell-to-cell communication called quorum sensing (QS) is mediated by chemical signal molecules^8^, and QS is known to be associated with biofilm maturation^9^. These findings are used to develop strategies to disrupt biofilm maturation using structural analogues of QS signal molecules (e.g. furanone, azithromycin, and 4-nitro-pyridine-N-oxide) or enzymes degrading QS molecules (e.g. acylase and lactonase). Some molecules have been shown to enhance cell dispersion during biofilm development. Enzyme dispersion B showing EPS degradation has been applied for this purpose^10^. Nitric oxide and cis-2-decenoic acid, an unsaturated fatty acid, are also reported to induce biofilm dispersion^7^.

Various natural products are effective in regulating biofilm development. The advantages of using natural products in biofilm inhibition are their lower toxicity and higher specificity compared to synthetic compounds^11^. Bean sprouts, chamomile, carrots, propolis, yellow peppers, water lilies, harbanero, garlic, fruit of a south-east Asian tree (*Lagerstroemia speciosa*), peas, medicinal plants from southern Florida (e.g. *Conocarpus erectus*), grapefruit, etc. have been shown to possess anti-QS activity^12^,^13^,^14^,^15^, and a few (e.g. garlic extract) show antibiofilm performance. We previously demonstrated that ginger extract had a capability to reduce biofilm formation for various bacteria including *P. aeruginosa* by reducing cellular cyclic diguanylate (c-di-GMP)^16^, but the active compound(s) associated with the finding has not been identified.

The goal of this study was to screen novel compound(s) of ginger extract that can inhibit *P. aeruginosa* biofilm formation, and explore the biofilm inhibition mechanism. We identified raffinose, a galactotrisaccharide, which was effective in reducing biofilm formation by attaching to a carbohydrate-binding protein and by controlling c-di-GMP that affects the initial and probably, the dispersal stages of biofilm development.

## Results

### Screening of biofilm inhibitors in ginger

**Figure 1:**
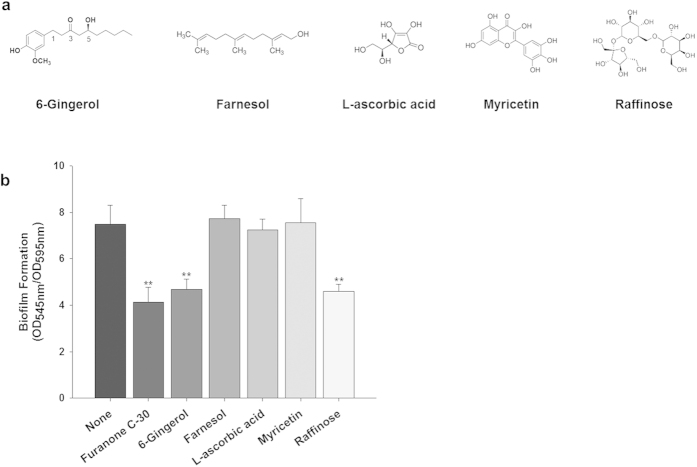
Screening of compounds from ginger inhibiting biofilm formation. (**a**) Putative ginger compounds used in this study. (**b**) Quantification of *P. aeruginosa* biofilm formation with the putative ginger compounds and furanone C-30 (a positive control) for 24 h in the wells of microtiter plates. The concentration of each compound was 10 μM. At least two independent experiments were conducted using 8 wells of microtiter plates, error bars indicate one standard deviations. **P < 0.0005 versus negative control.

Five putative ingredients frequently detected in ginger extract (6-gingerol, farnesol, L-ascorbic acid, myricetin, and raffinose, Fig. 1a) were tested for their potential to inhibit biofilm formation. Those ingredients were selected because they were identified in the ginger extract showing antibiofilm effect (data not shown). The degree of inhibition of biofilm formation by these candidates was evaluated using a static biofilm assay based on *P. aeruginosa* PA14 as a model bacterium in 96-well microtiter plates. Furanone C-30, a well reported biofilm inhibitor showing significant antibiofilm effect at 10 μM via QS inhibition^17^, was used as the positive control in this experiment. Figure 1b shows that 6-gingerol and raffinose reduced biofilm formation by 39% and 43%, respectively, while farnesol, L-ascorbic acid, and myricetin did not significantly reduce biofilm formation. The biofilm inhibiting effects of 6-gingerol and raffinose were comparable to furanone C-30 which showed 46% inhibition. Since the effect of 6-gingerol on biofilm inhibition has been previously discussed by us^18^, in this study, we report on the ability of raffinose to inhibit biofilm formation and the associated inhibitory mechanisms.

### Effects of raffinose on growth and biofilm formation

**Figure 2:**
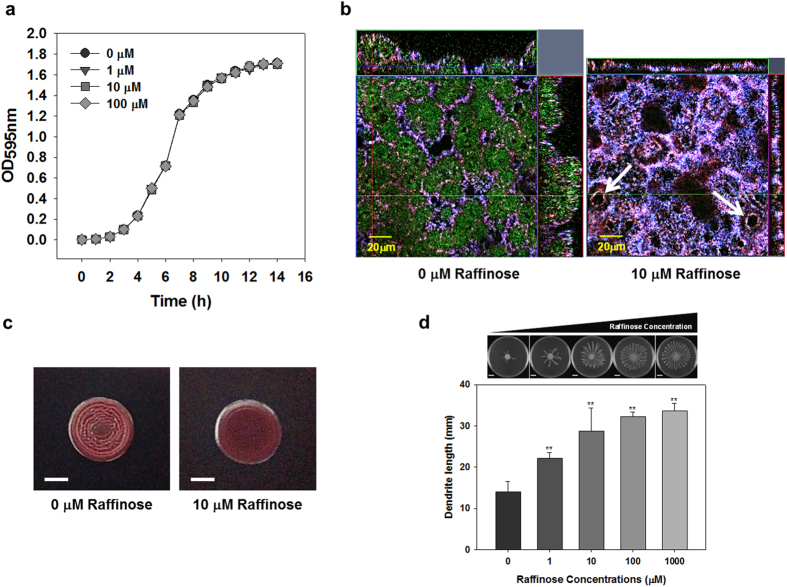
Effects of raffinose on *P. aeruginosa* phenotypes. (**a**) Growth at different concentrations of raffinose (0, 1, 10, and 100 μM) for 14 h incubation in flasks. Error bars indicate the standard deviations of 3 independent cultures. (**b**) 3-dimensional analyses of CLSM images of *P. aeruginosa* biofilm untreated (left), and with 10 μM raffinose treatment (right), on the glass slides. The biofilms were then stained using three fluorophores specific for nucleic acids (DAPI; blue), carbohydrates (ConA; green), and proteins (Ruby; red), before being subject to confocal laser scanning microscopy (CLSM) imaging. Arrows indicate hollow structure in the biofilm. (**c**) Colony morphology of *P. aeruginosa* without (left) and with 10 μM raffinose treatment (right) on Congo red agar plates. (**d**) Top images show swarming motility of *P. aeruginosa* cultured with different concentrations of raffinose (0, 1, 10, 100, and 1,000 μM) for 24 h on 0.5% BM-2 plate. Scale bars indicate 1 cm. Bottom graph shows the length of dendrites generated by swarming motility for each plate. **P < 0.0005 versus the control.

The growth of *P. aeruginosa* was measured for batch cultures with 0–100 μM raffinose. The growth curves for different raffinose concentrations were not significantly different in lag, exponential, and stationary phases during 14 h of incubation (Fig. 2a). The results suggested that the growth of *P. aeruginosa* was unaffected by the addition of raffinose up to 100 μM.

A static biofilm assay was performed with different concentrations of raffinose (0–1,000 μM). Biofilm formation was reduced gradually (17–58%) with increasing raffinose concentration (Fig. S1), demonstrating that raffinose inhibited biofilm formation in a concentration-dependent manner. The biofilm inhibition capacity of the raffinose’s hydrolyzed products of α-galactosidase (D-galactose and sucrose) was evaluated. α-galactosidase is widely distributed in microorganisms, plants, and animals^19^. D-galactose and sucrose did not reduce or increase the biofilm formation up to 1,000 μM concentration (Fig. S1). There were no statistical differences in biofilm formation between control and D-galactose or sucrose treatment (P > 0.05).

The effect of raffinose on biofilm formation was also tested in a flow reactor where growth medium with or without 10 μM raffinose was continuously dripped on glass slides for 24 hours. Figure 2b shows typical biofilms. The biofilm formed with raffinose treatment showed relatively lower thickness (8.4 μm) and smaller volume (4.8 μm^3^/μm^2^) than that without raffinose treatment (35.2 μm and 10.5 μm^3^/μm^2^, respectively). Although the biofilm formed without raffinose treatment demonstrated a typical *P. aeruginosa* mushroom morphology, the biofilm formed with 10 μM raffinose treatment was relatively flat. Interestingly, the biofilm with 10 μM raffinose treatment showed hollow structures (1,210 ± 170 per mm^2^) (see arrows in Fig. 2b) which were not evident in the biofilm without raffinose treatment.

### Effect of raffinose on the other phenotypes

Congo-red containing plates were used to observe *P. aeruginosa* colony morphology and to evaluate the effect of raffinose on exopolysaccharide production. Congo red is a pigment that binds to the exopolysaccharide matrix of bacterial biofilms, and leads to rugose colony morphology in *P. aeruginosa*^20^. As shown in Fig. 2c, a typical pink rugose morphology was evident for the colony without raffinose treatment, while the colony with raffinose treatment (10 μM) showed pink smooth morphology. This result is an indication of a reduction of exopolysaccharide production by raffinose treatment in *P. aeruginosa*.

The effect of raffinose on exopolysaccharide production was further evaluated by directly measuring total carbohydrate content in EPS from both planktonic and biofilm cells grown with 0–1,000 μM raffinose in growth medium. Exoprotein (a component of the biofilm matrix) production was also analyzed. Both planktonic and biofilm cells treated with raffinose significantly reduced the total carbohydrate and protein content of the EPS (Fig. S2). Total carbohydrate reduced by 21–53% and 22–48% (planktonic and biofilm cells, respectively), while total protein was reduced by 1–41% and 9–42% (planktonic and biofilm cells, respectively), in a dose-dependent manner, when treated with raffinose.

Biofilm formation in *P. aeruginosa* is reported to be inversely regulated by swarming motility^21^. Effect of raffinose (0–1,000 μM) on swarming motility was evaluated by growing *P. aeruginosa* cells on 0.5% soft agar plates and measuring the length of dendrites from the center (Fig. 2d). The dendrite lengths of the cells grown with raffinose were 1.6–2.4 fold longer than those grown without raffinose treatment and the length increased with increasing raffinose concentration. This suggests that raffinose promoted swarming motility on the soft agar plates. However, there were no changes in swimming or twitching motility by raffinose treatment (Fig. S3).

### Binding of raffinose to LecA

**Figure 3:**
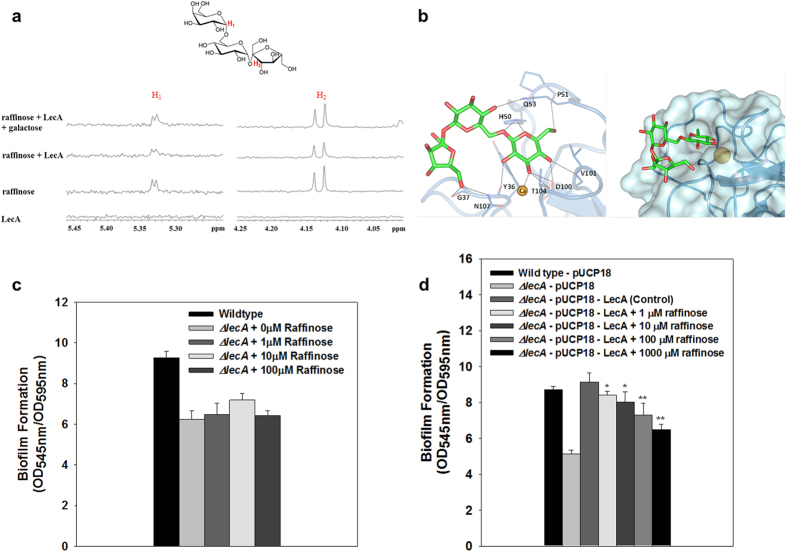
Interactions of raffinose and *P. aeruginosa* LecA protein. (**a**) Binding of raffinose to LecA monitored by CPMG NMR spectroscopy. First row: NMR spectrum of raffinose (0.1 mM) and LecA (50 μM) with the addition of galactose (1 mM) Second row: NMR spectrum of raffinose (0.1 mM) in the presence of LecA (50 μM). Third row: NMR spectrum of raffinose (0.1 mM) in the absence of LecA. Fourth row: NMR spectrum of LecA only. (**b**) *In silico* analysis of raffinose and LecA. Hydrogen-bonding interactions between LecA and the best-docked pose of raffinose (left). The docked raffinose on the surface of LecA (right). (**c**) Biofilm formation at different concentrations of raffinose (0, 1, 10, and 100 μM) in Δ*lecA* mutant for 24 h in microtiter plates. (**d**) Biofilm formation at different concentrations of raffinose (0, 1, 10, 100, and 1,000 μM) in Δ*lecA*-pUCP18-LecA for 24 h in microtiter plates. At least two independent experiments were conducted using 10 wells of microtiter plates, error bars indicate one standard deviations. **P < 0.0005 versus the control. *P < 0.005 versus the control.

*P. aeruginosa* produces two carbohydrate-binding proteins (lectins): LecA (PA-IL) and LecB (PA-IIL), which show a specificity for D-galactose and L-fucose, respectively^22^,^23^. Because LecA demonstrated high affinity to D-galactose and its derivatives^22^,^24^ as well as involvement in *P. aeruginosa* biofilm development^25^, it is reasonable to hypothesize that raffinose, a D-galactose derivative, may bind to LecA and thereby to suggest a mechanism for affecting *P. aeruginosa* biofilm development. To test this hypothesis, we initially evaluated the binding of raffinose to LecA based on 1D relaxation-edited nuclear magnetic resonance (NMR)-binding experiments^26^,^27^ using raffinose and LecA (Fig. 3a). NMR signals of 0.1 mM raffinose were monitored in the absence and presence of 50 μM LecA in buffer solution. In addition, 1 mM galactose was added to monitor the change of raffinose signals by competition at the same binding site. In the absence of LecA protein (third row spectra, Fig. 3a), indicative peaks for H_1_ and H_2_ signals of raffinose at 5.33 and 4.13 ppm were seen. These broadened and weakened in the presence of LecA protein (second row spectra, Fig. 3a), and the signal intensities recovered with the addition of a 10-fold excess of D-galactose (first row spectra, Fig. 3a). The results indicated that raffinose binds to LecA and its binding site overlaps with the galactose-binding site. Moreover, the signals for the protons at C-1 and C-6 of the galactose at 5.17 and 3.40 ppm in 50 mM LecA solution were also recovered by the addition of 1 mM raffinose (Fig. S4). Conversely, the galactose (0.1 mM) signals in LecA solution were also enhanced with treatment of a 10-fold excess of raffinose (Fig. S4). These results suggest that raffinose and galactose compete for the same site of the LecA protein.

To confirm the direct binding of raffinose to LecA, the binding affinities of raffinose, D-galactose, and sucrose to LecA were measured by isothermal titration calorimetry. Raffinose showed a 1.5-fold increase in binding (*K*_d_ = 32 μM) to LecA as compared with D-galactose (*K*_d_ = 47 μM), while sucrose which lacks the galactose moiety did not bind to LecA (Fig. S5), indicating that the galactose moiety of raffinose is a determinant of LecA binding. Binding enthalpies (Δ*H*), entropies (Δ*S*), and free energies (Δ*G*) of raffinose and D-galactose are summarized in Table S1.

To better understand the binding of raffinose to LecA, *in silico* docking studies of raffinose were performed using the X-ray structure of LecA in complex with melibiose (PDB: 4AL9). Most of the docked raffinose poses showed common binding patterns in which the galactose moiety was projected toward the calcium ion of the LecA active site, while the glucose and fructose moieties were oriented toward the surface and the loop region of LecA. The best-docked pose of raffinose is shown in Fig. 3b. The galactose moiety of the raffinose interacted strongly with Tyr 36, His 50, Pro 51, Asp 100, Val 101, and Thr 104. The glucose and fructose regions also interacted by hydrogen-bonding with Gln 53, Gly 37 and Asn 107, respectively.

Binding of raffinose to LecA and its relevance to biofilm inhibition were further verified using *P. aeruginosa* strains, one that cannot produce LecA and one that overproduces LecA. As shown in Fig. 3c, the *lecA* knockout mutant (a strain not producing LecA) demonstrated reduced biofilm formation and, furthermore, the biofilm formation did not decrease with increases in the raffinose concentration (P > 0.05). On the contrary, as shown in Fig. 3d, the *lecA* knockout mutant carrying pUCP18-LecA (a LecA overproducing strain) formed a biofilm similar to the wild-type strain carrying an empty vector (pUCP18). Additionally, the *lecA* knockout mutant carrying pUCP18-LecA showed a reduction in biofilm formation in the presence of raffinose, in a concentration-dependent manner (0–1,000 μM) (Fig. 3d). These results confirm that LecA is essential to form a biofilm, and suggested that raffinose binding to LecA is required for biofilm inhibition.

### Decrease in cellular cyclic diguanylate (c-di-GMP) production

**Figure 4:**
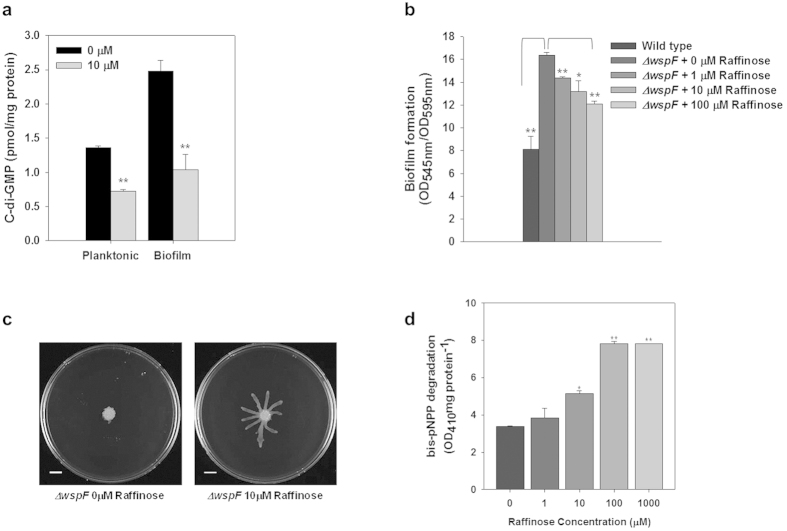
Effect of raffinose on cellular c-di-GMP levels. (**a**) Cellular c-di-GMP levels in *P. aeruginosa* planktonic and biofilm cells cultured without and with 10 μM raffinose for 24 h. Error bars indicate the standard deviations of 3 independent measurements. **P < 0.005 versus the control. *P < 0.05 versus the control. (**b**) Quantification of biofilm formation in *P. aeruginosa wspF* mutant with different concentrations (0, 1, 10, and 100 μM) of raffinose for 24 h in the wells of microtiter plates. At least two independent experiments were conducted using 8 wells of microtiter plates, error bars indicate one standard deviations. **P < 0.00005 versus the control. *P < 0.005 versus the control. (**c**) Swarming motility of *wspF* mutant cultured without (left) and with 10 μM raffinose (right) for 24 h on 0.5% BM-2 plate. (**d**) Degradation of the PDE-specific substrate bis-pNPP by *P. aeruginosa* cells cultured with raffinose (0, 1, 10, and 100 μM). **P < 0.000005 versus the control. *P < 0.00005 versus the control.

Cellular c-di-GMP plays important roles in the molecular determination between planktonic and sessile lifestyles, the regulation of motility, and the induction of virulence factor production^28^. In our previous study, we reported that ginger extract reduced the cellular c-di-GMP levels in both planktonic and biofilm cells of *P. aeruginosa*^16^. Accordingly, we evaluated the effect of raffinose on cellular c-di-GMP in both planktonic and biofilm cells. In general, biofilm cells exhibited higher c-di-GMP levels than planktonic cells (Fig. 4a), in agreement with other studies^29^. Furthermore, treatment with 10 μM raffinose reduced c-di-GMP levels in both planktonic (47%) and biofilm cells (59%).

In addition, to verify the relevance of raffinose treatment to cellular c-di-GMP regulation, a *P. aeruginosa* mutant which can overproduce c-di-GMP (Δ*wspF*) was used. The *wspF* gene in *P. aeruginosa* is reported to encode a methylesterase^30^. The methylesterase is presumed to control the activity of WspR protein which contains a CheY-like receiver and a diguanylate cyclase (GGDEF) domain^30^. Because Δ*wspF* shows constitutive activation of the WspR protein, Δ*wspF* leads to elevation of cellular c-di-GMP level and EPS production^31^. As shown in Fig. 4bΔ*wspF* induced around a 2-fold increase in biofilm formation compared to the wild-type strain. However, biofilm formation of Δ*wspF* was reduced 12–26% by raffinose treatment (0–100 μM), although the reduction of biofilm formation did not reach that of the wild type strain. This might be due to the fact that raffinose treatment was not sufficient to reduce cellular c-di-GMP level of Δ*wspF* mutant to the level of the wild type strain (Fig. S6). Interestingly, raffinose enhanced the swarming motility of Δ*wspF*. As shown in Fig. 4cΔ*wspF* showed limited swarming motility on a 0.5% soft agar plate, but Δ*wspF* grown with 10 μM raffinose clearly showed lengthened dendrites.

The above result appears to be related to a reduction of cellular c-di-GMP levels. One of the routes in reducing cellular c-di-GMP level is enhanced c-di-GMP specific phosphodiesterase (PDE) activity of *P. aeruginosa*. This possibility was tested by measuring the PDE activity, by analyzing the degradation of a PDE-specific substrate, bis-p-nitrophenol phosphate (bis-pNPP), in *P. aeruginosa* grown with raffinose. Figure 4d shows that *P. aeruginosa* grown with raffinose increased the activity of bis-pNPP degradation 1.1–2.3 fold, as compared with the same strain grown without raffinose. This result demonstrates that raffinose may reduce the *P. aeruginosa* cellular c-di-GMP levels via induction of PDE activity.

## Discussion

Besides biofilm reduction, raffinose altered other phenotypes of *P. aeruginosa*, such as inhibition of rugose colony formation in Congo-red plate (Fig. 2c), reduction of EPS production (Fig. S2), and increasing swarming motility (Fig. 2d). These altered phenotypes are typically observed when cellular c-di-GMP levels decrease 16,32. Therefore, it is reasonable to infer that raffinose triggered various phenotypic alternations by reducing cellular c-di-GMP. This speculation is confirmed by the experiments using a *P. aeruginosa* mutant that overproduces cellular c-di-GMP (*ΔwspF*): raffinose reduced biofilm formation (Fig. 4b) concomitant with recovery of swarming motility (Fig. 2d).

C-di-GMP levels are regulated by two enzymes acting oppositely. C-di-GMP is produced from two GTPs by diguanylate cyclases (DGCs) and is degraded by phosphodiesterases (PDEs) into two GTPs via pGpG^33^. Two routes are possible for decreasing cellular c-di-GMP levels: decreasing the activity of DGCs with GGDEF domain or increasing the activity of PDEs with EAL and/or HD-GYP domains. The experimental results show that increasing concentrations of raffinose increase the PDE activity of *P. aeruginosa* (Fig. 4d), suggesting that the latter route was the mechanism decreasing c-di-GMP levels by raffinose. This hypothesis is supported by the study of Chung *et al.*^31^ who increased PDE activity by cloning *pvrR*, a PDE gene, in a plasmid vector using the *P. aeruginosa ΔwspF* mutant. The transformed *ΔwspF* mutant showed increased PDE activity along with decreased c-di-GMP levels and enhanced swarming motility. In explaining the decrease in c-di-GMP level by raffinose treatment, the possibility of a decrease in the activity of DGCs could not be excluded because of the lack of supporting evidence leaving the mechanism open.

On the other hand, a reduced level of cellular c-di-GMP is reported to be associated with the motile mode of growth and dispersal as well as reduced biofilm formation^32^. Biofilm dispersal is initiated by localized cell death and lysis, to provide nutrients for escaping cells 7. This entails hollowing of biofilm microcolonies^34^. In this study, hollow structures in the biofilm treated with raffinose (Fig. 2b) suggest that raffinose triggers biofilm dispersion as well as reduction in biofilm formation by reducing cellular c-di-GMP level. This speculation was further evaluated by measuring released planktonic cells from *P. aeruginosa* biofilms formed on glass slides. The number of released cells increased by 1.8–3.6 fold when treated with 100 μM raffinose, as compared with untreated cells (Fig. S7). Taken together, our data demonstrating the regulation of cellular c-di-GMP levels by raffinose have enhanced our understanding about galactosides binding to LecA and their inhibition of *P. aeruginosa* biofilm formation (i.e., LecA-ligand competitive inhibition was not the sole mechanism for biofilm inhibition). Nevertheless, it is not clear whether LecA-raffinose binding caused the decrease in c-di-GMP or raffinose decreased c-di-GMP independent from LecA-raffinose binding, which will be investigated in the near future.

It is also important to note that LecA production is related to biofilm formation. Diggle *et al.*^25^ observed *lecA* gene expression in a *P. aeruginosa* biofilm, and showed enhanced biofilm formation by a LecA-overproducing strain, and reduction by a *lecA* mutant. We investigated a possible interference of *lecA* gene expression by raffinose treatment using RT-qPCR analyses (Fig. S8). The expression of the *lecA* gene was reduced by 50% in the wild type *P. aeruginosa* by raffinose treatment. Interestingly, the *P. aeruginosa* mutant overproducing cellular c-di-GMP (Δ*wspF*) demonstrated a 1.5-fold increase in *lecA* gene expression over the wild-type strain, with a 30% decrease in *lecA* gene expression after raffinose treatment. Furthermore, the *P. aeruginosa lecA* knockout mutant (Δ*lecA*) showed a decrease in cellular c-di-GMP levels and a 1.5-fold increase in the activity of c-di-GMP specific PDE as compared with the wild-type strain. These results imply that binding of raffinose to LecA reduced *lecA* gene expression and in turn, decreased cellular c-di-GMP levels, which resulted in biofilm inhibition. Nevertheless, detailed molecular mechanisms should be investigated for a better understanding about LecA-mediated biofilm formation.

Raffinose is a common galactooligosaccharide found in various vegetables (e.g. beans, cabbage, and broccoli) and plant seeds^35^, as well as in ginger. It is not clear why some plants produce bacterial biofilm inhibitors such as raffinose. It would be interesting to study whether such biofilm inhibitors are produced accidentally or via evolutionary processes. Whatever the reason, the production of biofilm inhibitors appears to provide survival benefits for the plants. For example, Austrian red algae are reported to produce halogenated furanones effective in inhibiting biofilm formation^17^, which avoids colonization by microorganisms that, in turn, block light to the plant. Biofilm inhibitors could be potentially applied in various medical and industrial settings. Increasing numbers of antibiotic-resistant pathogenic bacteria cause difficulties in antibiotic therapy. For example, more than 95% *P. aeruginosa* strains are reported to be resistant to gentamicin, carbenicillin, co-trimoxazol, ceftizoxime, and tetracycline 3. Biofilm inhibitors that do not affect bacterial growth are an emerging alternative to antibiotics in treating infectious diseases involving biofilm formation. They can also reduce biofouling problems, replacing biocides used in membrane filters for water treatment, water distribution pipes, ship hulls, laundry machines, humidifiers, dishwashers, etc. We believe that the findings from this study will be helpful to design more potent LecA-mediated biofilm inhibitors for combating unwanted biofilms.

## Experimental Procedures

### Bacterial strains and chemicals

*P. aeruginosa* PA14 was used as a model biofilm-forming bacterium. *wspF* or *lecA* knockout mutant of *P. aeruginosa*^36^ was used to test whether raffinose was also effective for bacteria that overproduce cellular c-di-GMP or could not produce LecA. Ginger’s ingredients, such as 6-gingerol, farnesol, L-ascorbic acid, myricetin, and D-(+)-raffinose pentahydrate, were purchased from Sigma Aldrich (St. Louis, MO, USA), and were dissolved in dimethyl sulfoxide (Carl Roth, Karlsruhe, Germany).

### Growth inhibitory test

Overnight culture of *P. aeruginosa* was grown in AB medium (300 mM NaCl, 50 mM MgSO_4_, 0.2% vitamin-free casamino acids, 10 mM potassium phosphate, 1 mM L-arginine, and 1% glucose, pH 7.5)^37^ with varying concentrations of raffinose (0, 1, 10, or 100 μM) using a shaking incubator maintained at 37 °C, 250 rpm, for 14 h. Bacterial growth was evaluated by measuring optical density (OD) at 595 nm using a UVmini-1240 spectrophotometer (Shimadzu, Kyoto, Japan) in triplicate samples collected hourly.

### Static biofilm formation assay

Overnight cultures of test bacteria (OD at 595 nm = 1.5) were diluted in fresh AB medium (1:20) and aliquoted into wells of a TPP® 96-well polystyrene microtiter plates (Sigma Aldrich). After forming biofilms in the wells at 37 °C without agitation for 24 h, OD of suspended cultures was measured at 595 nm using an iMark microplate absorbance reader (BioRad, Richmond, CA, USA), after which the suspended cultures were discarded. The plate was washed with phosphate-buffered saline (PBS) (137 mM NaCl, 2.7 mM KCl, 10 mM Na_2_HPO_4_, and 2 mM KH_2_PO_4_, pH 7.2) to remove remaining suspended cells. The biofilms were stained with 1.0% crystal violet for 30 min, and then washed with autoclaved deionized water to remove remaining unbound dye. The crystal violet bound to biofilms was eluted by 100% ethanol and OD of the eluent was measured at 545 nm. The biofilms were quantified as the amount of crystal violet bound to biofilms (OD at 545 nm), normalized by the amount of suspended cells (OD at 595 nm).

### Biofilm formation assay using a drip-flow reactor

One ml of overnight culture of *P. aeruginosa* (OD at 595 nm = 1.5) was diluted in 19 ml fresh AB medium (1:20) in a petri dish. A glass slide was dipped into the petri dish containing the dilution and incubated at 37 °C for 24 h to form a biofilm on the glass slide. The glass slide covered with biofilm cells was inserted into the DFR-110 drip-flow biofilm reactor (BioSurface Technologies, Bozeman, MT, USA). Fresh AB medium without or with raffinose (10 μM) was continuously fed into the reactor using a peristaltic pump (Masterflex C/L tubing pumps, Cole-Parmer, Vernon Hills, IL, USA) at 20 ml∙h^−1^ and operated in an incubator at 37 °C for 24 h. Then, the glass slide was carefully washed with PBS (pH 7.2). The attached biofilm cells were stained with DAPI (Carl Roth), FITC-labeled ConA (Sigma Aldrich), and SYPRO Ruby (Invitrogen, Carlsbad, CA, USA) sequentially for 20 min each. The stained biofilm cells were washed twice with PBS (pH 7.2) after each staining step. DAPI, ConA, and Ruby solutions were targeted to stain DNA, carbohydrate, and protein, respectively. The biofilm cells were then observed using CLSM (Carl Zeiss LSM700, Jena, Germany). Confocal images were taken with a 40× objective lens (C-Apochromat 40×/1.20 W Korr M27, Carl Zeiss), and the images of blue (DAPI), green (ConA), and red (RUBY) fluorescence were observed simultaneously with the Z-stack mode using the Zen 2011 program (Carl Zeiss). Detailed methods about the fluorescent filters are described in a previous study^16^. Bio-volume (μm^3^/μm^2^), biofilm average thickness (μm), and roughness coefficient were measured by Comstat2 in the ImageJ program^38^.

### Construction of a pUCP-LecA expression plasmid

LecA protein was ectopically expressed using a multicopy plasmid, pUCP18^39^. The sequence of the *lecA* gene region was amplified by PCR using Pfu polymerase (PfuUltra II Fusion HS DNA Polymerase, Agilent Technologies, Santa Clara, CA, USA) with the following oligonucleotides: 5′- ATGGCCGGATCCCGTTGCTGTGCTTTGCTG (BamHI restriction site is underlined) and 5′- CAGAAGCTTCGGCCACACGGGCAACTTCAC (HindIII restriction site is underlined). The amplified 540-bp fragment and plasmid pUCP18 were digested with both BamHI and HindIII endonucleases, gel purified, and ligated using T4 ligase (Promega, Madison, WI, USA). The ligated plasmid was transformed into *Escherichia coli* (XL10-Gold Ultracompetent cells, Agilent Technologies), as per the manufacturer’s protocol.

### Transformation of *P. aeruginosa* with pUCP-LecA expression plasmids

The preparation of competent cells for *P. aeruginosa* wild type and Δ*lecA* mutant, and the transformation of *P. aeruginosa* using an electroporation method, were based on a previous study^40^. 1.5 mL of overnight culture of *P. aeruginosa* wild type and Δ*lecA* cells grown in LB medium, were harvested by centrifugation at room temperature for 3 min at 16,000× g. The cell pellet was washed twice using 1 mL of 300 mM sucrose, and then the cell pellet was resuspended in 100 μL of 300 mM sucrose. For electroporation, purified plasmid DNA (pUCP18 and pUCP-LecA) using the Qiagen plasmid prep kit (Qiagen, Chatsworth, CA, USA) was mixed with 100 μL of *P. aeruginosa* competent cells. The purified plasmid DNA pUCP18 (empty vector) was mixed with wild type and Δ*lecA* competent cells, while the purified pUCP-LecA was mixed with Δ*lecA* competent cell. The mixture was transferred to a 0.2 cm gap width electroporation cuvette (BIO-RAD, Hercules, CA, USA) and pulsed (25 μF, 200 Ω, 2.5 kV) using the Gene Pulser Xcell^TM^ electroporation system (BIO-RAD). The electroporated cells (100 μL) were transferred to a 15-ml conical tube, 1 mL of fresh LB medium was added, and the mixture was shaken at 250 rpm at 37 °C for 1 h. The cells were spread on an LB plate with 100 μg/mL carbenicillin and incubated at 37 °C for 14 h. Colonies were picked and grown in LB broth. Plasmid DNA was isolated, treated with BamHI and HindIII endonucleases, and separated using gel electrophoresis, to confirm insertion of the *lecA* gene into the plasmid.

### Colony morphology assay

Overnight culture of *P. aeruginosa* was diluted in fresh T-broth medium (10 g/L tryptone) with raffinose (0 or 10 μM), and the dilution was incubated using a shaking incubator at 37 °C, 250 rpm for 14 h (OD at 595 nm = 1.0). 2 μL of the culture was spotted on a Congo-red plate^16^, and incubated at room temperature for 3–7 days.

### EPS analysis

EPS of planktonic and biofilm cells was analyzed using a modified sonication method^16^. Planktonic cells were prepared by diluting an overnight culture of *P. aeruginosa* in fresh AB medium (1:20) with 0–1,000 μM raffinose, and incubated on a shaking incubator at 37 °C, 250 rpm, overnight. Biofilm cells were prepared using borosilicate bottles by diluting overnight cultures of *P. aeruginosa* in fresh AB medium (1:20) with raffinose (0–1,000 μM) and by incubating the dilution at 37 °C for 24 h without agitation. OD of the suspended cells from both planktonic and biofilm cultures were measured at 595 nm using a spectrophotometer. The biofilm cells were resuspended in 3 mL 0.01 M KCl by vortexing. Planktonic and biofilm cultures were centrifuged at 8,000× g and the harvested cells were resuspended in 10 mL and 3 mL 0.01 M KCl, respectively. The resuspended cells were disrupted using a sonicator (VCX 750, SONICS, Newtown, CT, USA) for 4 cycles of 5 s of operation and 5 s of pause at a power level of 3.5 Hz. The sonicated cells were centrifuged at 4,000× g, and the supernatants were filtered through 0.22-μm Millex filter (Car Roth). Filtered supernatants were used for the quantification of protein and carbohydrate. Protein analysis was performed by the Lowry method in 96-well microtiter plate: 40 μL filtrate and 200 μL Lowry reagent (L3540, Sigma Aldrich) were mixed in a microtiter plate, and the mixture was incubated for 10 min at room temperature, after which 20 μL Folin-Ciocalteu reagent (F9252, Sigma Aldrich) was added to the mixture, and the mixture was incubated for 30 min. Protein quantity was measured by measuring OD at 750 nm using the iMark microplate reader. Carbohydrate was analyzed by phenol-sulfuric acid method in a 96-well microtiter plate: 15 μL filtrate and 150 μL sulfuric acid were mixed in a microtiter plate, and the mixture was incubated for 30 min at room temperature: 30 μL 5% phenol was added to the mixture, and the mixture was incubated at 90 °C in a water bath. Carbohydrate quantity in the mixture was measured by measuring OD at 490 nm using the iMark microplate reader. Protein (OD at 750 nm) and carbohydrate (OD at 490 nm) quantity were normalized by suspended cells (OD at 595 nm).

### Swarming motility assay

Swarming motility was assayed based on a previous method^16^. 10 μL overnight cultures of *P. aeruginosa* with raffinose (0–1,000 μM) was spotted on a BM-2 plate (62 mM potassium phosphate [pH 7.0], 2 mM MgSO_4_, 10 mM FeSO_4_, 1% casamino acid, 0.4% glucose, and 0.5% Bacto agar) and incubated at 37 °C for 24 h. The degree of swarming motility was evaluated by measuring average length of dendrites.

### RT-qPCR analysis

RT-qPCR was performed to quantify and compare the levels of *P. aeruginosa lecA* gene expression. The *lecA* and housekeeping gene *proC* primer sets were designed using Primer 3 version 0.4.0 (http://frodo.wi.mit.edu/) (Table S2). Total RNA extraction was performed from *P. aeruginosa* biofilm cells using TRI REAGENT (Molecular Research Center, OH, USA), following the manufacturer’s instruction. RT-qPCR was performed using the Bio-Rad CFX-96 real time system (Bio-Rad, Hercules, CA, USA). The reaction mixture consisted of the following reagents: 10 μL SYBR Premix Ex Taq^TM^ (Takara, Shiga, Japan), 0.8 μL each of the forward and reverse primers (10 μM), 0.4 μL of 50 X ROX^TM^ Reference Dye I, 2 μL extracted total RNA (100 ng/ml), and RNase free water, to generate a 20 μL final volume. Detailed protocols about RNA extraction and RT-qPCR methods are described in our previous study^18^.

### Biofilm dispersion assay

An overnight culture of *P. aeruginosa* was diluted in fresh AB medium (OD at 595 nm = 0.05). A polystyrene petri dish was filled with 20 mL of the dilution. A sterilized glass slide was dipped into the petri dish and incubated at 37 °C for 24 h to develop biofilms on the glass slide. The slide was then gently rinsed using PBS (pH 7.2), and dipped into the petri dish containing 20 mL of AB medium with or without 100 μM raffinose. The petri dish was incubated for 2 h and 4 h at 37 °C, respectively. 100 μL of the culture was serially diluted to 10^−5^ in PBS (pH 7.2). For counting colony forming units, 100 μL of the final dilution was spread on LB agar plates and incubated overnight at 37 °C.

### Statistical analysis

*P*-values for testing statistical differences between measurements were estimated by a student’s *t*-test (Excel software, Microsoft, Redmond, WA, USA).

### NMR analysis

All spectra were measured at 298 K on a Bruker Avance 600 MHz spectrometer (Billerica, MI, USA). The 1D relaxation-edited NMR experiments utilized CPMG pulse sequences with excitation sculpting for water suppression. The pre-acquisition delay was 1.2 s, and total spin lock time was 400 ms. The delay for spin echo between successive 180 pulses in CPMG experiment was 2 ms. The data were collected with a sweep width of 16 ppm (9,600 Hz) and 128 scans. Data processing was performed with Topspin 3.0 (Bruker) software with a sine square window function over 16384 complex data points before Fourier transformation. Samples contained 50 μM LecA protein (L9895, Sigma Aldrich) and 0.1 mM of raffinose or galactose in a 10% D_2_O buffered solution. For the displacement experiment, 1 mM of competitor (raffinose vs. galactose) was added to each sample.

### *In silico* docking studies

*Ligand preparation and optimization:* Raffinose was generated as 2D and 3D structures by *ChemBioDraw* (ver. 11.0.1) and *Chem3D Pro* (ver. 11.0.1), respectively, being saved in *.mol* file format. The process of ligand preparation and optimization was performed by the *‘Sanitize’* preparation protocol in *SYBYL-X 2.1.1* (Tripos Inc., St Louis, MI, USA).

*Protein preparation:* The protein structures of lectin PA-IL from *P. aeruginosa* in PDB format were downloaded from the RCSB protein data bank (PDB ID: 4AL9). A structure preparation tool in *SYBYL-X* 2.1.1 was employed for protein preparation. The crystal ligand melibiose, water molecules, Ca^2+^ ion, and other chains except chain F were removed from the protein-ligand complexes for docking. Conflicted side-chains of amino acid residues were fixed. Hydrogen atoms were added under the application of *TRIPOS* Force Field as a default setting. A minimization process was performed by the *POWELL* method, the initial optimization option was changed to *None* from a default setting *SIMPLEX,* and the termination gradient and max iteration were set to 0.5 kcal/(mol*Å) and 1000 times, respectively. After protein minimization, processed LecA protein structure was saved as *.mol2* file format.

*Docking and scoring function studies:* The docking study of raffinose was performed by *Surflex-Dock GeomX* module in *SYBYL-X 2.1.1*. Docking was guided by the *Surflex-Dock* protomol. Protomol, containing information on the binding site of Lectin PA-IL for *Surflex-Dock*, was defined according to the location information of the original crystal ligand melibiose. Two factors related with the generation of Protomol are *Bloat(Å)* and *Threshold* which were set to 0.5 and 0, respectively. The maximum number of generated poses per each ligand and the minimum RMSD between final poses were set to 20 and 0.05, respectively. Other parameters were applied with its default settings in all runs.

### Cellular c-di-GMP concentration analysis

For the analysis of c-di-GMP in planktonic cells, 4.9 mL of 37% formaldehyde (Carl Roth) was added to one liter of overnight cultures of *P. aeruginosa* (OD at 595 nm = 1.5) grown without or with raffinose (10 μM). The cultures were centrifuged at 8,000× g for 10 min, and the harvested cells were resuspended in 40 mL PBS (pH 7.2) containing 0.18% formaldehyde. For the analysis of c-di-GMP in biofilm cells, initially, an overnight culture of *P. aeruginosa* was diluted in fresh AB medium (1:20). The dilution was aliquoted into borosilicate bottles without or with raffinose (10 μM) and incubated at 37 °C for 24 h without agitation. After decanting the suspended cells, the biofilm cells were washed twice with PBS (pH 7.2). Biofilm cells were resuspended by vortexing in 3 mL PBS containing 0.18% formaldehyde. Planktonic and biofilm cells were centrifuged at 8,000× g for 10 min, and the harvested cells were resuspended in 5 mL deionized water. The resuspensions were boiled for 10 min, and cooled in ice for 10 min. The cooled resuspensions were mixed with 100% ethanol (9.3 mL) for extraction of nucleotides, and then the mixtures were centrifuged at 8,000× g for 10 min at 4 °C. The supernatants were filtered through a 0.22-μm filter (Car Roth, Karlsruhe, Germany) for analyzing cellular c-di-GMP. The harvested cells were used to analyze total protein using the Lowry method described above. The filtrated supernatants were lyophilized using Savant SpeedVac (SC201A, Thermo Scientific, San Jose, CA, USA), and then the lyophilized cells were dissolved in 200 μL buffer A (100 mM KH_2_PO_4_, 4 mM tetrabutyl ammonium hydrogen sulfate, pH 5.9). The dissolved solutions were filtered through 0.45-μm filter (Car Roth). Filtered solutions (20 μL) were analyzed using high-performance liquid chromatography (HPLC) (Hewlett Packard 1100 Series, Agilent, Santa Clara, CA, USA) with an ultraviolet detector (254 nm), and a Kinetex C-18 column (4.6 × 250 mm, 5 u, Phenomenex, Torrance, CA, USA) with mixing buffer (75% of buffer A, and 25% of pure methanol) as the mobile phase at the Korea Basic Science Institute (Seoul, Korea). C-di-GMP separation was achieved using a gradient HPLC method as follow: 0–4 min; 50:50, 5–20 min; 0:100, and 21–30 min; 50:50 (operational time (min); ratio of buffer A and methanol) at a flow rate of 0.8 mL∙min^-1^.

### *In vitro* PDE activity assay

*In vitro* PDE activity was assayed based on a previous study^29^. Overnight cultures of *P. aeruginosa* were diluted in fresh AB medium (1:100), treated with varying raffinose concentrations (0–1,000 μM), and incubated in a shaking incubator at 37 °C, 250 rpm for 14 h. The suspended cultures were centrifuged at 6,000× g for 10 min at 4 °C, and the harvested cells were resuspended in 1 ml of PDE activity assay buffer (50 mM NaCl, 50 mM Tris base [pH 8.1], 1 mM MnCl_2_, and 5 mM bis-pNPP). Bis-pNPP (N3002, Sigma Aldrich) used as a synthetic substrate of c-di-GMP specific PDE. The resuspended cells were lysed using CelLytic Express (C5491, Sigma Aldrich). For the PDE reaction, the mixture was incubated at 37 °C for 2 h in a shaking incubator at 60 rpm. The OD of the mixture was then measured at 410 nm using an iMark microplate absorbance reader for analyzing bis-pNPP degradation into *p*-nitrophenol. PDE activity (OD at 410 nm) was normalized by total proteins measured by the Lowry method, as described above.

## Additional Information

**How to cite this article**: Kim, H.-S. *et al.* Raffinose, a plant galactoside, inhibits *Pseudomonas aeruginosa* biofilm formation via binding to LecA and decreasing cellular cyclic diguanylate levels. *Sci. Rep.* **6**, 25318; doi: 10.1038/srep25318 (2016).

## Supplementary Material

Supplementary Information
