# Supplementary material for: Raffinose, a plant galactoside, inhibits Pseudomonas aeruginosa biofilm formation via binding to LecA and decreasing cellular cyclic diguanylate levels
Source: Sci Rep. 2016 May 4;6:25318. doi: 10.1038/srep25318 (PMC4855137; doi:10.1038/srep25318)
Supplement: Supplementary Information [file srep25318-s1.pdf]

Raffinose, a plant galactoside, inhibits *Pseudomonas aeruginosa* biofilm formation via binding to LecA and decreasing cellular cyclic diguanylate levels

Han-Shin Kim[1], Eunji Cha[1], YunHye Kim[2], Young Ho Jeon[2], Betty H. Olson[3], Youngjoo Byun[2*] and Hee-Deung Park[1*]
1-School of Civil, Environmental and Architectural Engineering, Korea University, Anam-Dong, Seongbuk-Gu, Seoul 136-713, South Korea
2-College of Pharmacy, Korea University, Sejong-ro 2511, Jochiwon-eup, Sejong, 339-700, South Korea
3-Department of Civil and Environmental Engineering, University of California, Irvine, CA 92697, USA

Figure S1

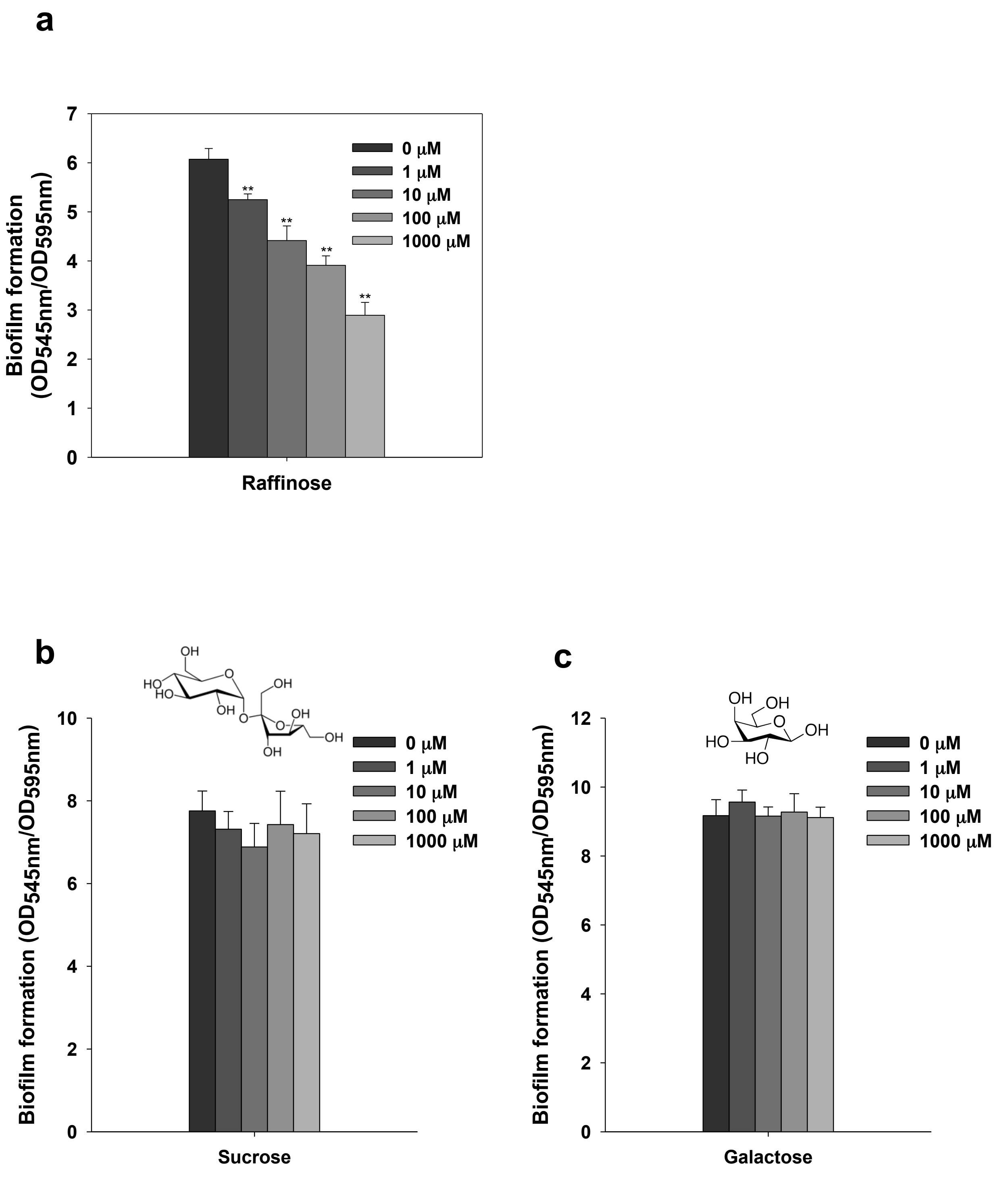

Fig. S1. Effects of raffinose on *P. aeruginosa* biofilm formation. Biofilm formation at different concentrations (0, 0.1, 1, 10, 100, and 1000 µM) of raffinose (a), sucrose (b) , and D-(+)-galactose (c) for 24 h in microtiter plates. Error bars indicate the standard deviations of 10 measurements. **, P < 0.0005 versus the control. *, P < 0.005 versus the control.

Figure S2

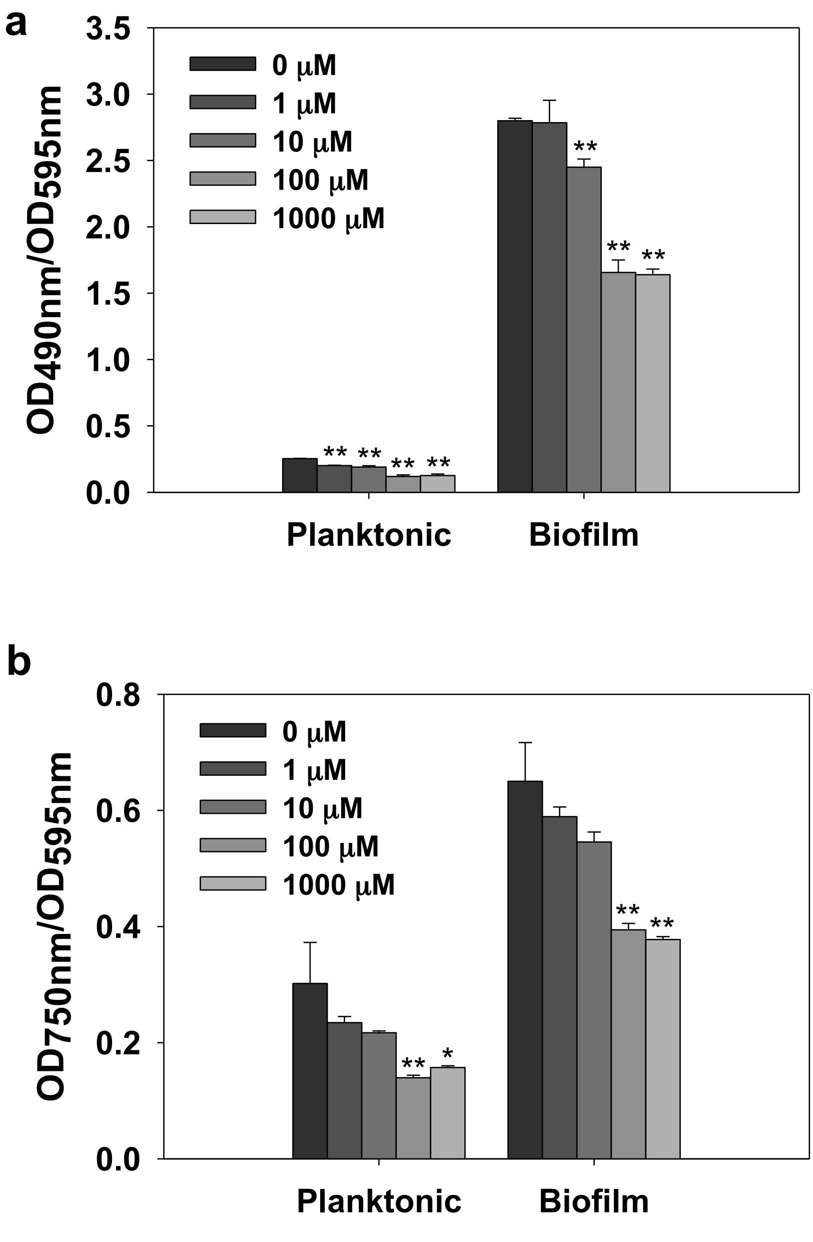

Fig. S2. Effect of raffinose on EPS production. (a) Total carbohydrate in EPS of *P. aeruginosa* planktonic and biofilm cells cultured with different concentration of raffinose (0, 1, 10, 100, and 1,000 μM) for 24 h. (b) Total protein in EPS of *P. aeruginosa* planktonic and biofilm cells cultured with different concentration of raffinose (0, 1, 10, 100, and 1,000 μM) for 24 h. Error bars indicate the standard deviations of 3 measurements. **, $P < 0.005$ versus the control. *, $P < 0.05$ versus the control.

Figure S3

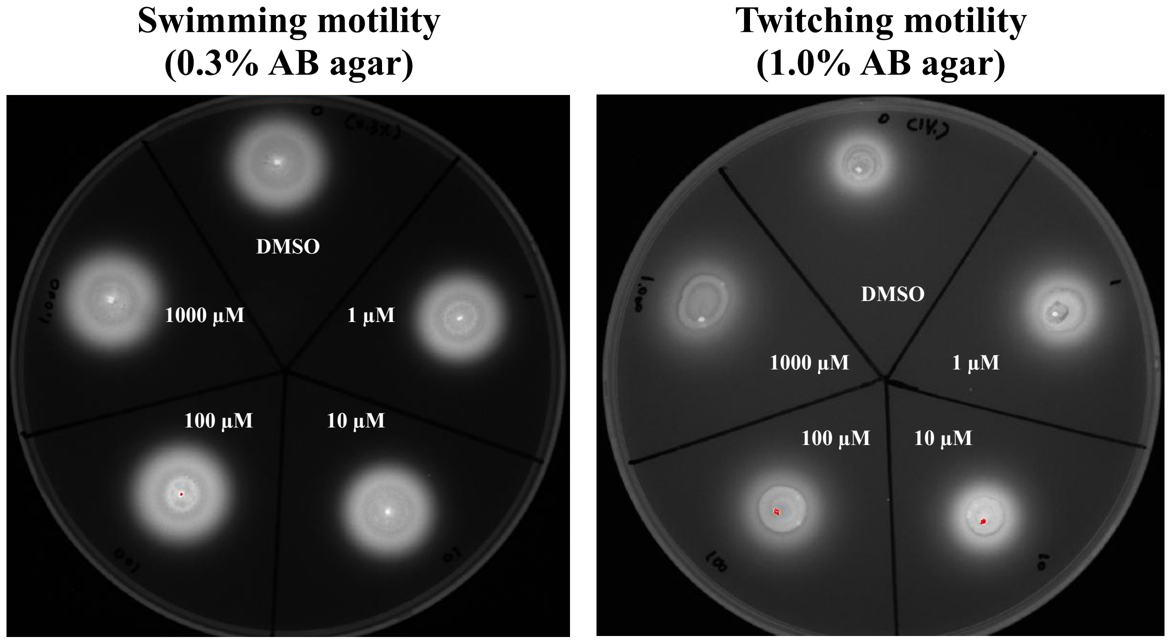

Fig. S3. Swimming and twitching motility of *P. aeruginosa* cultured with different concentrations of ra ffinose (0, 1, 10, 100, and 1,000 μM) for 24 h on AB agar plate.

Figure S4

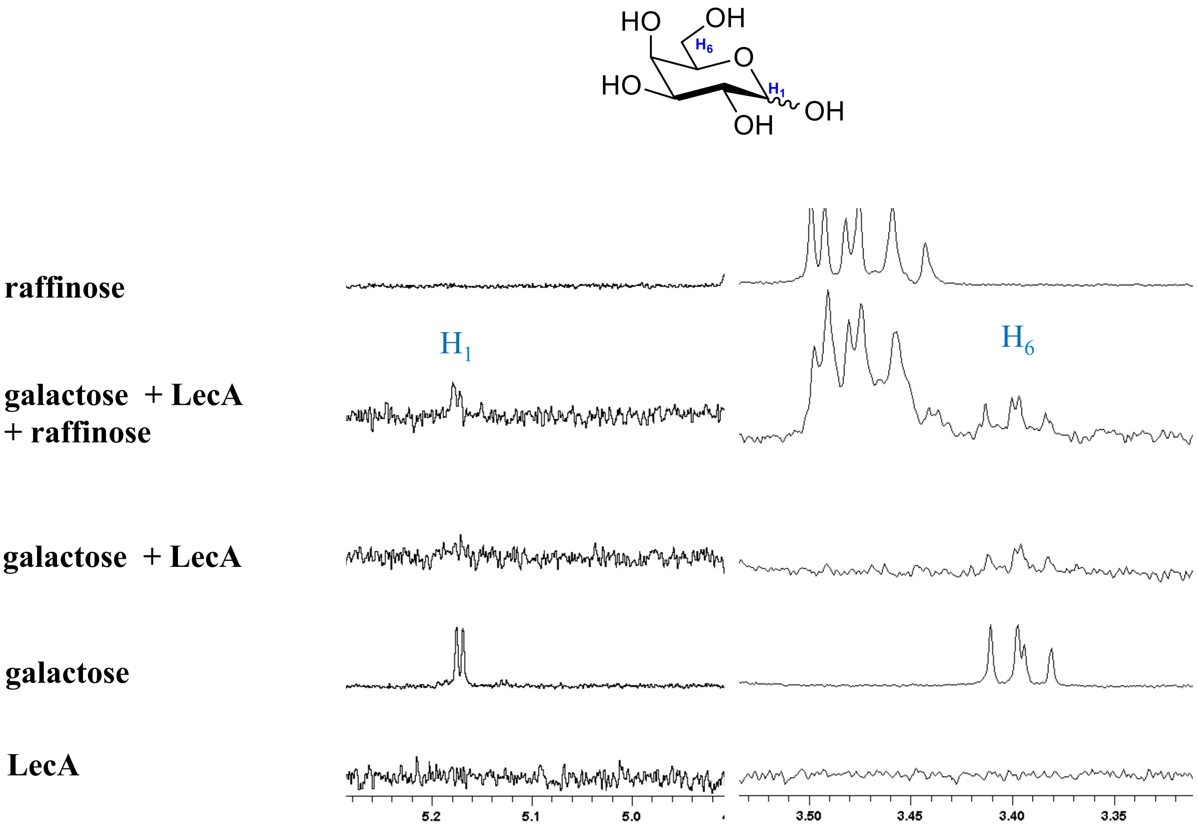

Fig. S4. Monitoring of galactose signals with the treatment of excess raffinose by CPMG NMR spectroscopy. First row: NMR spectrum of raffinose in the absence of LecA. Second row: NMR spectrum of galactose (0.1 mM) and LecA (50 µM) with the addition of raffinose (1 mM). Third row: NMR spectrum of galactose (0.1 mM) in the presence of LecA (50 µM). Fourth row: NMR spectrum of galactose (1 mM) in the absence of LecA. Fifth row: NMR spectrum of LecA only

Figure S5

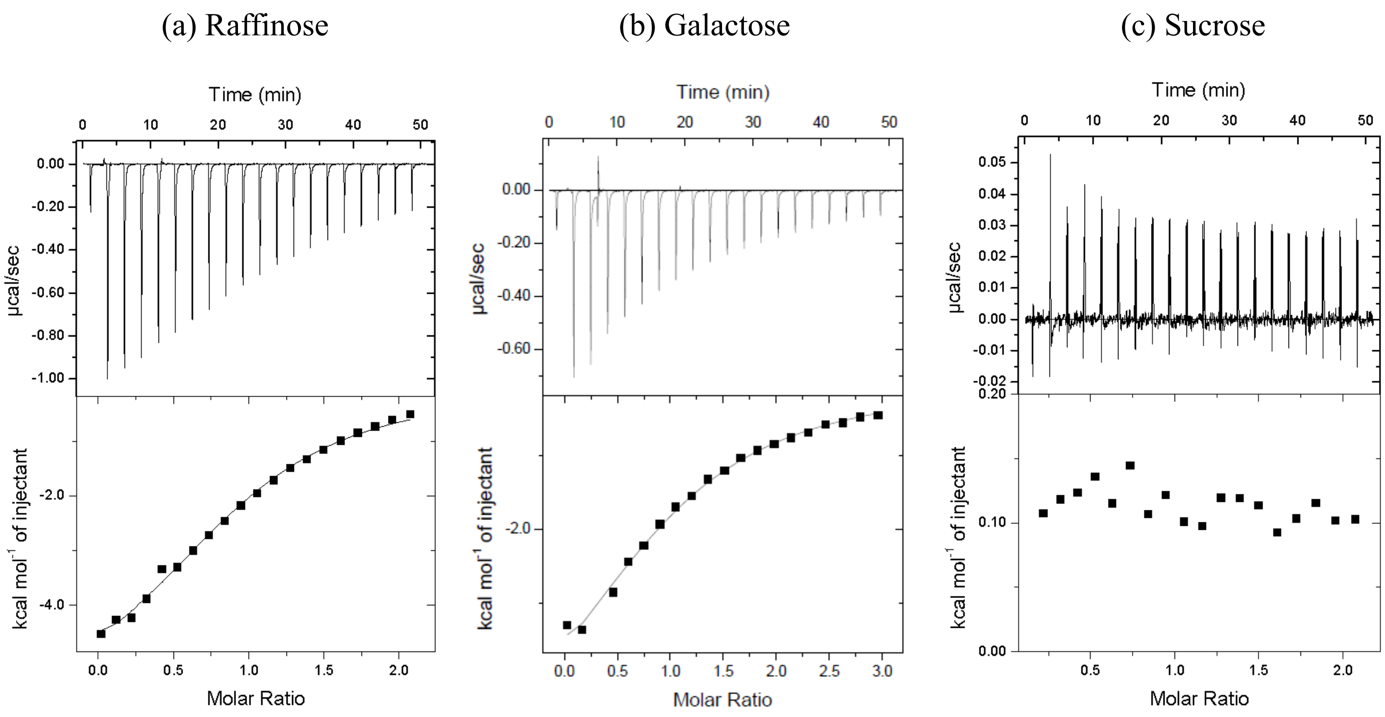

Fig. S5. Isothermal titration calorimetric (ITC) measurements for the binding of the ligands to LecA. Th e raw ITC raw data (first row) and integrated titration curves (second row) of raffinose, galactose, and s ucrose.

Figure S6

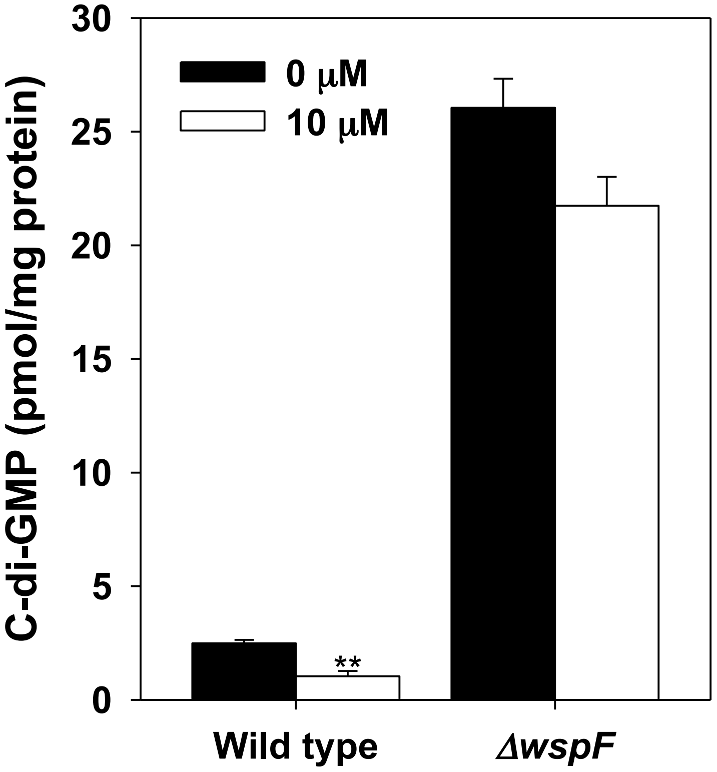

Fig. S6. Effects of raffinose on cellular c-di-GMP levels in *P. aeruginosa*. Cellular c-di-GMP levels in *P. aeruginosa* wild type and *ΔwspF* mutant treated without and with 10 μM raffinose for 24 h. Error ba rs indicate the standard deviations of 3 measurements. **, P < 0.0005 versus the control.

Figure S7

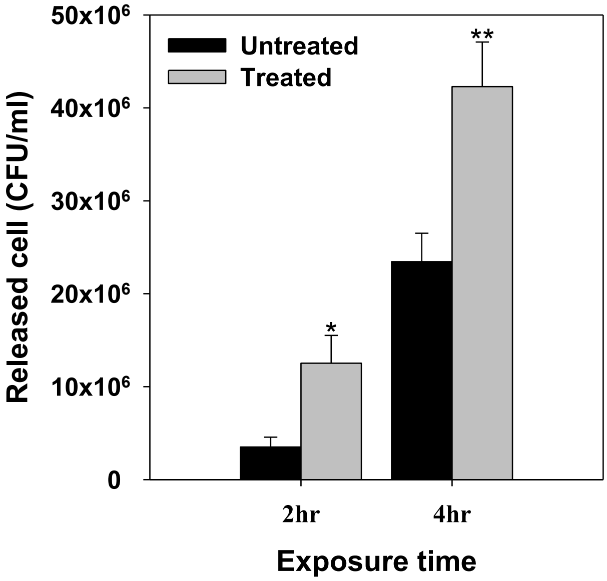

Fig. S7. Effects of raffinose on *P. aeruginosa* biofilm dispersion. Bacterial cells formed biofilm format ion on the glass slide and 100 μM concentrations of raffinose treats for 2 and 4 h. Released bacteria cel l count assay. Error bars indicate the standard deviations of 3 measurements. **, P < 0.0005 versus the control. *, P < 0.005 versus the control.

Figure S8

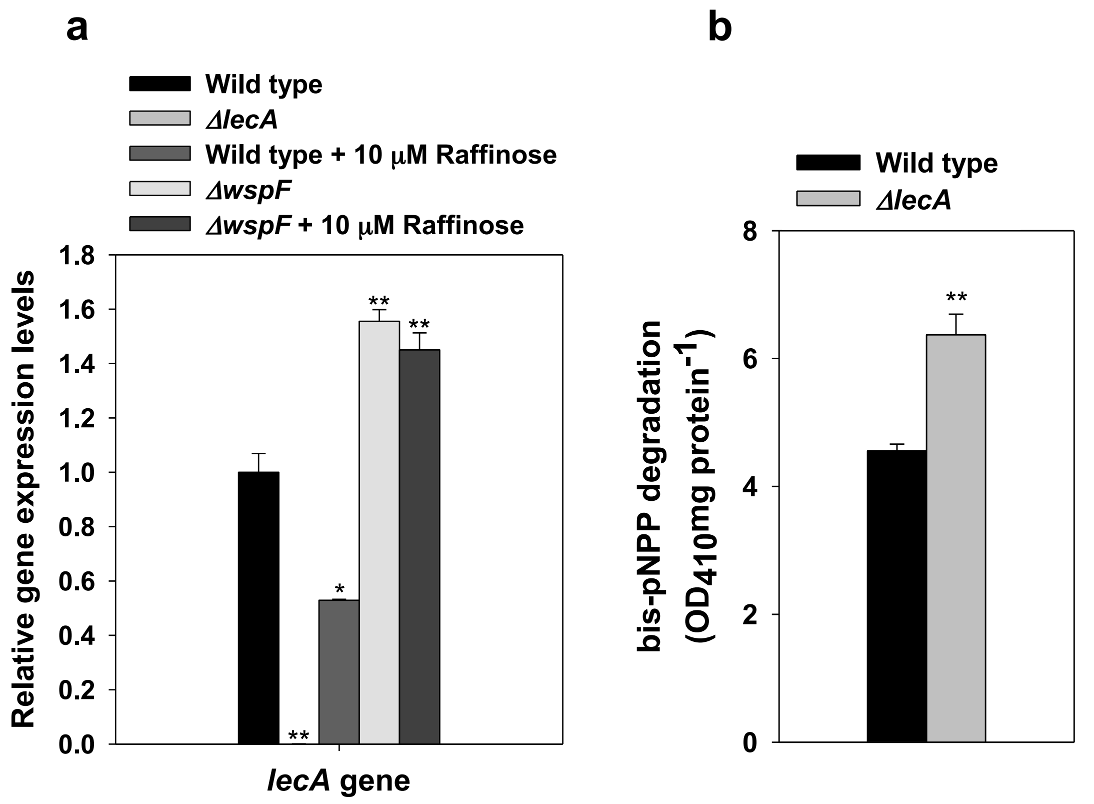

Fig. S8. Effects of raffinose on *P. aeruginosa lecA* gene expression and PDE activity. (a) Relative *lecA* gene expression for *lecA* deletion mutant (*ΔlecA*) and c-di-GMP overproducing mutant (*ΔwspF*) with a nd without addition of 10 μM raffinose. (b) PDE activity measurement via bis-PNPP degradation for *le cA* deletion mutant (*ΔlecA*). Error bars indicate the standard deviations of 3 measurements. **, P < 0.0 005 versus the control. *, P < 0.005 versus the control.

Table S1

Table S1. Thermodynamic parameters and K_d values of raffinose and galactose from ITC experiments.

Ligand	ΔH (kcal/mol)	-TΔS (kcal/mol)	ΔG (kcal/mol)	Stoichiometry	K_d (μM)
Raffinose	-6.02	-0.11	-6.13	0.95	32
Galactose	-5.74	-0.16	-5.90	1.01	47

Table S2

Table S2. RT-qPCR primers used in this study.

Primer	Target gene	Sequence (5’→3’)	Tm (^{o}C)	GC %	Product size (bp)	Reference
lecA-F	*lecA*	GGG TTG CAC CCA ATA ATG TC	57.3	50	100	This study
lecA-R	*lecA*	CCA ATA TTG ACG CTG AAC GA	55.3	45		
proC-F	*proC*	GGC GTA TTT CTT CCT GCT GA	60.4	50	236	Savli H et al.
proC-R	*proC*	CCT GCT CCA CTA GTG CTT CG	61.2	60		

Savli H, *et al.* (2003) Expression stability of six housekeeping genes: A proposal for resistance gene quantificat ion studies of *Pseudomonas aeruginosa* by real-time quantitative RT-PCR. *J Med Microbiol* 52(Pt 5): 403-408.
